# Validation of A Nationwide Digital Pediatric Pathology Consultation Network

**DOI:** 10.1177/10935266251316782

**Published:** 2025-02-10

**Authors:** Haiying Chen, Juan Putra, Anita Nagy, Jefferson Terry, Dina El Demellawy, Joseph de Nanassy, Erica Schollenberg, Aaron Haig, Camelia Stefanovici, Kathryn Whelan, Alysa Poulin, Dorothee Dal Soglio, Zesheng Chen, Brian Smith, Cindy Fiore, Gino R. Somers

**Affiliations:** 1 Division of Pathology, The Hospital for Sick Children, Toronto, ON, Canada; 2 Department of Laboratory Medicine and Pathobiology, University of Toronto, ON, Canada; 3 Anatomical Pathology, BC Children's Hospital, Vancouver, BC, Canada; 4 Department of Pathology and Laboratory Medicine, Faculty of Medicine, University of British Columbia, BC, Canada; 5 Department of Pathology, The Children’s Hospital of Eastern Ontario, ON, Canada; 6 Department of Pathology and Laboratory Medicine, Faculty of Medicine, University of Ottawa, Ottawa, ON, Canada; 7 Division of Anatomical Pathology, IWK Health Centre, NS, Canada; 8 Department of Pathology, Dalhousie University, NS, Canada; 9 Department of Pathology, London Health Sciences Centre, ON, Canada; 10 Pathology and Laboratory Medicine, Western University, ON, Canada; 11 Department of Pathology, Max Rady College of Medicine, University of Manitoba, MB, Canada; 12 Department of Pathology, Health Sciences Centre, Winnipeg, MB, Canada; 13 Faculty of Medicine, Memorial University, St. John's, NL, Canada; 14 Pathology and Laboratory Medicine, Royal University Hospital, Saskatchewan Health Authority, Saskatoon, SK, Canada; 15 College of Medicine, University of Saskatchewan, Saskatoon, SK, Canada; 16 Department of Pathology, CHU-St. Justine, Montreal, QC, Canada; 17 Faculty of Medicine, University of Montreal, Montreal, QC, Canada; 18 Centre for Global Child Health, The Hospital for Sick Children, Toronto, ON, Canada

**Keywords:** digital pathology, whole slide imaging, pediatric, nationwide, consultation, validation

## Abstract

**Background:**

Digital pathology facilitates remote pathology consultations. Pediatric pathologists in Canada formed a nationwide digital pathology consultation network, mostly for second opinion review of pediatric cancer cases. Validation of such a large network for clinical use is challenging. Here we report our unique validation process of this digital pathology network.

**Method:**

This study was designed in keeping with the College of American Pathologist (CAP) guidelines, and included 14 pathologists from 9 hospitals across Canada. All cases are pediatric pathology cases. Each pathologist reviewed multiple digital cases and the corresponding glass slide cases. For each review, intra-observer concordance (diagnosis on digital case versus diagnosis on glass slide case) was recorded, creating a data point.

**Result:**

The study generated 269 valid diagnostic data points. Out of the 269 data points, 257 were concordant (95.5% concordance), exceeding the CAP recommendation of 95% concordance. Thus, the network was successfully validated.

**Conclusion:**

This is a unique validation study for a large nationwide digital pediatric pathology network. The study involved all pathologists/hospitals in the network, closely emulating real world clinical process. The network was successfully validated.

## Introduction

Pediatric cancers are uncommon. In Canada, approximately 880 children under the age of 15 are diagnosed with cancer each year.^1^ Diagnosing pediatric cancer can be challenging for both general and pediatric pathologists, and due to its rarity, second-opinion pathology consultations are very important in this field. Canada is a large country with a relatively small population. Large pediatric academic centers and subspecialty expertise are scattered in different parts of the country, making second-opinion consultation difficult. The routine process of an external pathology consultation requires packaging and mailing glass slides to an expert pediatric pathologist in another hospital, often in another city or province. This process is costly, time-consuming and is associated with the risk of damaging or losing original glass slides.

To improve the efficiency of remote consultations, pediatric pathologists in 9 hospitals across Canada formed a nationwide digital pathology network (Canadian Digital Paediatric Pathology Network, or CDPPN), an internet-based telepathology platform using whole-slide imaging (WSI) for remote consultations. The 9 hospitals are in 7 Canadian provinces, spanning coast to coast, covering most of the pediatric population in Canada. To our knowledge, this is the first-ever nationwide digital pediatric pathology consultation network. This article focuses on the process used for validation of this network for clinical use.

WSI is the cornerstone for digital pathology and has been approved by both Health Canada and the United States Food and Drug Administration (FDA) for routine pathology use.^2,3^ To ensure patient safety, digital pathology systems should be validated before clinical use. The College of American Pathologists (CAP) published guidelines for the validation of WSI systems in 2013, with updates published in 2022.^4,5^ In the most recent CAP guideline (2022), the main recommendations for a successful validation study included a sample set of at least 60 cases, an intra-observer concordance (same pathologist, using digital slides versus using glass slides) of at least 95%, and a washout period of at least 2 weeks between viewing the glass slides and the digital slides.^5^ The validation of CDPPN followed the most recent CAP guidelines.

## Materials and Methods

### Implementation of the CDPPN Network

Since January 2021, 14 Canadian pediatric pathologists from 1 initiating hospital and 8 partner hospitals have been meeting online regularly to form a nationwide digital pathology consultation network, CDPPN. Each participating hospital uses its own Health Canada-approved WSI scanner to produce digital slides. The scanners used included Aperio AT2 (Leica), Aperio LV1 IVD (Leica), Aperio AT Turbo (Leica), NanoZoomer S360 (Hamamatsu), and Panoptiq (ViewsIQ). All slides were scanned at 20× (equivalent to using 20× objective of a light microscope). TRIBUN, a global leader in digital pathology Cloud solutions, was chosen to provide the online platform. TRIBUN designed a secure website^6^ for CDPPN and provided training for all participating pathologists. The computer monitors used to view the online images included Lenovo (ThinkVision) T2424pA, Dell C2423H, Dell D175, HP Z30i, Dell U2412Mb, Dell T5820, NEC Multisync LCD 2190UXi, and ViewSonic VP3881.

The following is the basic workflow of a digital consultation: The pathologist requesting the consultation (the applicant) scans and uploads a digital case to the website. The applicant selects one or more consultation pathologists (the reviewer) and submits the request to the reviewer. The reviewer receives the request in real time (via text message to reviewer’s cell phone) and decides to either accept or reject the request. During the initial contact, the applicant and the reviewer can exchange opinions by text messaging on the website. If the case is accepted, the reviewer usually can provide a diagnosis (or a preliminary diagnosis) within a few days. Both informal and formal (pathology report issued) consultations are possible.

### Validation Study, Summary

**Figure 1.**
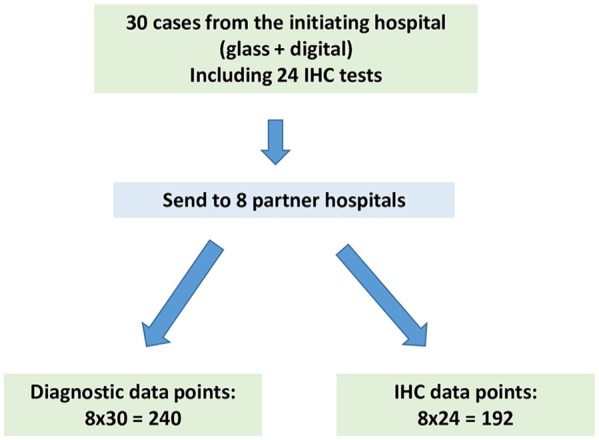
Schematic of part 1 of the validation process.

**Figure 2.**
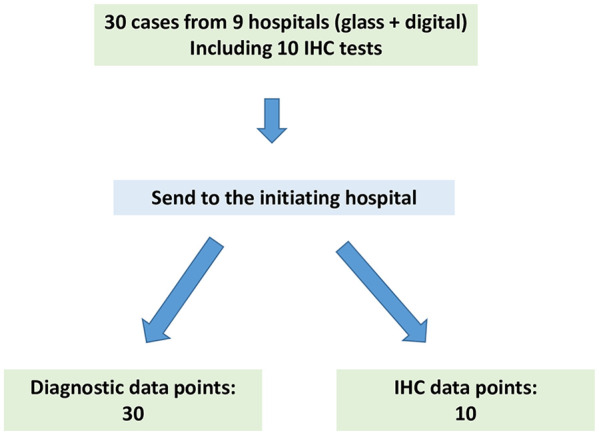
Schematic of part 2 of the validation process.

This project was approved by the participating hospitals as per their local policies. In the initiating hospital, this project was approved as a Quality Improvement Project. Research Ethic Board review was not required by the hospital. As per the CAP guidelines, validation studies should mimic the real-world clinical environment as closely as possible. To that end, our validation study involved the entire network and consisted of 3 parts. In part 1, 30 cases were chosen from the initiating hospital, and sent to the 8 partner hospitals for review (Figure 1). In part 2, an additional 30 cases were contributed by all participating hospitals and were reviewed at the initiating hospital (Figure 2). Some cases included immunohistochemistry (IHC) stains. The reviewers were also requested to interpret each IHC test (positive versus negative) as a separate result. Part 3 was data analysis. The following details each part of the validation process.

### Validation Study, Part 1

Thirty (30) pediatric cancer cases (accessioned from 2014 to 2018) were selected from the pathology archive of the initiating hospital. The cases included both common and rare pediatric cancers (Table 1). The cases were anonymized. One representative paraffin block was chosen from each case. Serial sectioning of the paraffin block was performed to produce at least 9 hematoxylin and eosin (H&E) stained glass slides, 1 slide for each participating hospital. When necessary, IHC stains were performed on additional serial sections of the block. All glass slides were reviewed by a pediatric pathologist at the initiating hospital to ensure that the original diagnostic features remained. In the end, 9 sets of glass slides were prepared, 1 for each participating hospital. Each set included 30 cases, with each case containing 1 to 3 glass slides (1 H&E slide and up to 2 IHC slides). The glass slide set for the initiating hospital was scanned into WSI files (digital slides), creating 30 digital cases.

**Table 1.** Cases for Part 1 of the Validation Process.

Case	Gender	Age (years)	Clinical information	Immunohistochemistry provided	Original diagnosis
1	F	6	Left kidney mass	N/A	Wilms tumor, triphasic with anaplasia
2	F	1	Left thigh soft tissue mass	BAF47	Proximal epithelioid sarcoma
3	F	11	Cervical lymphadenopathy	TdT	Acute lymphoblastic leukemia/lymphoma
4	F	4	Right kidney mass	N/A	Wilms tumor, no anaplasia
5	M	2	Left kidney mass	N/A	Biphasic Wilms tumor, with anaplasia
6	F	2	External genital polypoid lesion	Myogenin	Embryonal rhabdomyosarcoma
7	M	4	Left kidney mass	N/A	Post-treatment neuroblastoma, with differentiating neuroblastoma histomorphology
8	F	1	Posterior mediastinal mass	N/A	Differentiating neuroblastoma
9	F	3	Retroperitoneal mass	N/A	Ganglioneuroblastoma
10	M	2	Right nasal mass	Myogenin	Embryonal rhabdomyosarcoma
11	F	13	Cervical lymphadenopathy	CD30, Pax5	Classic Hodgkin lymphoma, nodular sclerosis type
12	F	1	Paraspinal mass	N/A	Neuroblastoma, poorly differentiated type
13	M	4	Nasopharyngeal mass	Myogenin	Embryonal rhabdomyosarcoma
14	M	13	Right hemi pelvic mass	CD99	Small round cell tumor, favor Ewing sarcoma
15	M	2	Right kidney mass	BAF47	Rhabdoid tumor
16	M	3	Right kidney mass	N/A	Wilms tumor, triphasic, no anaplasia
17	M	5	Left kidney mass	N/A	Wilms tumor, triphasic, no anaplasia
18	M	8	Maxillary sinus mass	CD99	Ewing sarcoma
19	M	9	Metastatic lung nodule	Myogenin	Metastatic embryonal rhabdomyosarcoma
20	F	8	Cervical lymphadenopathy	CD30, Pax5	Classic Hodgkin lymphoma, nodular sclerosis type
21	M	12	Cervical lymphadenopathy	CD30, Pax5	Classic Hodgkin lymphoma, nodular sclerosis type
22	F	11	Right neck mass	CD30, Pax5	Classic Hodgkin lymphoma, nodular sclerosis type
23	M	10	Left hand hypothenar mass	Myogenin	Alveolar rhabdomyosarcoma
24	M	5	Left kidney mass	N/A	Wilms tumor
25	F	7	Left kidney mass	N/A	Papillary renal cell carcinoma
26	M	4	Left neck mass	Myogenin	Rhabdomyosarcoma
27	M	2	Retroperitoneal mass	N/A	Ganglioneuroblastoma
28	F	9	Cervical lymphadenopathy	CD30, Pax5	Classic Hodgkin lymphoma, nodular sclerosis type
29	F	10	Left tibial mass	CD99	Ewing sarcoma
30	M	17	Cervical lymphadenopathy	CD30, Pax5	Classic Hodgkin lymphoma, nodular sclerosis type

Abbreviation: N/A, not applicable.

Next, 8 sets of the glass slides (30 cases in each set) were mailed to the 8 partner hospitals along with a brief clinical history, creating a total of 240 glass slide test cases. A pediatric pathologist in the partner hospital was asked to review the glass slides and provide a diagnosis as specific as possible based on the material received. A separate interpretation of each IHC test was also requested.

Meanwhile, the digital cases at the initiating hospital were randomized. After at least 2 weeks from the time of the glass slide review (washout time of at least 2 weeks), the digital cases were sent to all 8 partner hospitals via the CDPPN website, creating a total of 240 digital test cases. The same pathologist who reviewed the glass slide cases reviewed the digital cases and provided a diagnosis in the same manner as the glass slide case review.

### Validation Study, Part 2

In part 2, 30 cases were contributed from all the participating hospitals, with each hospital contributing 3–4 cases. Most were pediatric cancer cases, but non-cancer cases (such as placenta pathology and fetal autopsy cases) were also included (Table 2). Each case consisted of 1–4 glass slides which included IHC slides when applicable. These glass slides were scanned at the originating hospitals creating a total of 30 digital test cases. These 30 digital cases (with brief clinical histories) were uploaded to the CDPPN. Two pathologists in the initiating hospital reviewed the 30 digital cases in the same manner as for part 1, with each pathologist reviewing 15 cases.

**Table 2.** Cases for Part 2 of the Validation Process.

Case	Gender	Age (years)	Clinical information	Immunohistochemistry provided	Original diagnosis
1	M	13	Pancreatic mass	N/A	Pancreatic neuroendocrine tumor
2	Not provided	0 (fetus of gestational age 26 weeks)	Adrenal gland from autopsy	N/A	Adrenal cortical cytomegaly
3	F	33	Term placenta	N/A	Fetal thrombotic vasculopathy
4	M	14	Right leg lytic bone lesion, biopsy	CD45, ERG, CD99	Ewing sarcoma
5	F	0 (7 weeks old )	Right leg soft tissue mass	Myogenin, vimentin	Infantile fibrosarcoma
6	F	14	Mass lesion in left tibia	N/A	Osteosarcoma
7	F	16	Bone lesion, left scapula	N/A	Aneurysmal bone cyst
8	F	6	Ovarian mass with liver mets	N/A	Yolk sac tumor
9	F	3	Kidney mass	N/A	Wilms tumor
10	M	11	Mass lesion in tibia	N/A	Osteoid osteoma
11	F	15	Right fallopian tube cyst	N/A	Serous cystadenoma
12	F	5	Bone lesion, right iliac crest	N/A	Favor neuroblastoma
13	F	5	Scalp lesion	N/A	Neuroblastoma
14	F	17	Bone tumor	N/A	Conventional osteosarcoma
15	M	2	Liver mass	N/A	Hepatoblastoma
16	M	3	Kidney mass	N/A	Wilms tumor
17	F	11	Ovarian mass	N/A	Immature teratoma
18	F	34	Placenta, thick meconium noted at delivery	N/A	Chronic villitis
19	M	17	Bilateral breast masses	N/A	Gynecomastia
20	M	9	Femur cortical lesion	N/A	Non-ossifying fibroma
21	F	12	Ovarian mass	N/A	Yolk sac tumor
22	M	6	Sphenoidal mass	Myogenin	Embryonal rhabdomyosarcoma
23	M	1	Hepatic mass	N/A	Hepatoblastoma
24	M	6	Epiploic mass	N/A	Burkitt lymphoma
25	M	11	Abdominal mass	CD30, PAX5	Diffuse large B-cell lymphoma
26	F	0 (1 month old)	Left kidney mass	N/A	CMN, classic type
27	M	1	Mass in right abdomen	N/A	Mesenchymal hamartoma
28	F	13	Right distal femoral lesion	N/A	Osteosarcoma
29	F	6	Nasopharyngeal tumor	Desmin	Rhabdomyosarcoma
30	M	11	History of Ewing sarcoma; new pleural effusion; Please review H&E and IHC slides of cell block.	CD99	Ewing sarcoma

Abbreviation: N/A, not applicable.

At the same time, the glass slides of these cases (with brief clinical histories) were sent to the initiating hospital creating 30 glass slide test cases. These cases were randomized at the initiating hospital. After at least 2 weeks from the reviewing of digital slides (washout time of at least 2 weeks), the same pathologist who reviewed the digital cases reviewed the corresponding glass slide cases, using the same interpretive guidelines.

### Validation Study, part 3

Data analysis was performed at the initiating hospital. The first step of data analysis was to confirm data validity. A valid data point was defined as an intra-observer comparison of a valid digital pathology diagnosis versus a valid glass slide diagnosis of the same case. In part 1, a single digital pathology diagnosis was invalid due to illegible handwriting and could not be used for data analysis. Of the total of 192 possible IHC data points, only 144 data points were valid because some pathologists did not provide separate IHC result interpretation. In part 2, all 30 diagnostic data points were valid and only 5 out of 10 possible IHC data points were valid (same reason as for part 1).

Concordance grading was then performed. Each data point was put in 1 out of 3 concordance categories (Table 3). The grading was performed independently by 2 pediatric pathologists who were not one of the case reviewers in parts 1 and 2. For most of the data points the 2 grading pathologists assigned the same grade. For only a few data points the 2 grading pathologists assigned different grades. In these few occasions, a third (more senior) pediatric pathologist was asked to be a “tie breaker” (choosing one of the initial grades). Thus, all final grades were a consensus of at least 2 pediatric pathologists.

**Table 3.** Three Concordance Categories.

Concordance category	Definition
Concordant	Consistent or compatible
Minor discrepancy	Discrepant, but unlikely to alter clinical management
Major discrepancy	Discrepant, likely to alter clinical management

## Results

In part 1, out of the 239 valid diagnostic data points, 228 (95.4%) were concordant, 8 (3.3%) showed minor discrepancy, and 3 (1.3%) showed major discrepancy. In part 2, out of the 30 valid diagnostic data points, 29 (96.6 %) were concordant, 1 data point (3.3%) showed minor discrepancy, and none of the data points showed major discrepancy. There was a total of 149 valid IHC data points from 24 cases (parts 1 and 2 combined). All valid IHC date points were concordant (100% concordant).

The 2 validation steps generated a total of 269 valid diagnostic data points. 257 (95.5%) were concordant, 9 (3.3%) showed minor discrepancy, and 3 (1.1%) showed major discrepancy. The concordance rate for diagnostic data points was 95.5%, above the 95% cutoff recommended by CAP, thus the digital system was successfully validated.

There were a total of 12 discrepant data points which were further analyzed (Table 4). Feedback was given to the reviewers by email. The primary purpose of this validation study was to compare the digital slide diagnosis against the glass slide diagnosis. However, some interesting findings emerged when we compared the original diagnoses to the 2 new diagnoses. As shown in Table 4, in 7 discrepant data points, the digital slide diagnoses were closer to the original diagnoses. In 4 discrepant data points, the glass slide diagnoses were closer to the original diagnoses. For 1 discrepant data point (Table 4, case #23), both diagnoses were different from the original diagnosis. For the 3 data points with major discrepancy, the digital slide diagnoses were closer to original diagnoses in 2 of them. Thus, although the sample size was small, the performance of digital pathology was not inferior to glass slide pathology in this study if using the original diagnosis as a gold standard.

**Table 4.** Analysis of Discrepant Data Points.

Part	Case #	Original dx	Glass slide dx	Digital slide dx	Grade of discrepancy	Which one is closer to original dx?
1	5	Wilms tumor	CCSK	Possible Wilms tumor	Major	Digital slide
1	5	Wilms tumor	Wilms tumor	CCSK	Major	Glass slide
1	7	Treated NB, with differentiating NB morphology	GNB	Treated NB	Minor	Digital slide
1	7	Treated NB, with differentiating NB morphology	GNB, if post-chemo, then it is viable neuroblastic tumor	GN	Minor	Glass slide
1	7	Treated NB, with differentiating NB morphology	Pheochromocytoma	GNB	Major	Digital slide
1	9	Treated NB, with differentiating NB morphology	NB	GNB	Minor	Glass slide
1	9	Treated NB, with differentiating NB morphology	GNB	NB	Minor	Digital slide
1	13	ERMS	RMS, favor alveolar type	Small blue round cell tumor, favor ERMS	Minor	Digital slide
1	21	Classic Hodgkin lymphoma	Lymphoma	Classic Hodgkin lymphoma	Minor	Digital slide
1	23	ARMS	ERMS	Sclerosing RMS	Minor	Cannot decide
1	26	ERMS	ERMS	ARMS	Minor	Glass slide
2	27	Mesenchymal hamartoma	Ductal plate malformation	Favor mesenchymal hamartoma	Minor	Digital slide

Abbreviations: ARMS, alveolar rhabdomyosarcoma; CCSK, clear cell sarcoma of kidney; Dx, diagnosis; ERMS, embryonal rhabdomyosarcoma; GN, ganglioneuroma; GNB, ganglioneuroblastoma; NB, neuroblastoma; RMS, rhabdomyosarcoma.

## Discussion

One of the important applications for digital pathology is remote consultation, which is especially true for Canada, where only a few academic large pediatric centers are scattered across a large geographic area. This was the impetus for creating the nationwide digital pediatric pathology consultation network in Canada. WSI-based digital pathology is being rapidly accepted by the pathology community. Based on results of a multicenter study^7^ that included 1992 cases, the United States FDA approved the use of digital pathology for primary pathology diagnosis.^3^ Al-Janabi et al^8^ showed that WSI can be used for primary diagnosis of pediatric specimens. Compared to traditional glass slide microscopy, digital pathology has numerous advantages, including reduced risk of glass slide damage, improved and streamlined remote access, better data archiving, and the ability to integrate with artificial intelligence and digital image analysis, with comparatively few drawbacks such as increased cost and process complexity.

It is recommended that a digital pathology system be validated before clinical use.^5^ The most recent CAP guidelines^5^ include 3 strong recommendations and 9 good practice statements (GPSs) for validation. The 3 strong recommendations are (1) using at least 60 cases for validation, (2) an overall intra-observer concordance of at least 95%, and (3) using a washout period of at least 2 weeks. The 9 GPSs are (1) all laboratories carrying out their own validation studies, (2) validation appropriate for the intended clinical use, (3) validation closely emulating the real world clinical environment, (4) testing the entire WSI system, (5) preparing for possible changes to the WSI system that could impact clinical results, (6) training pathologists for WSI use, (7) ensuring all material on a glass slide scanned to the digital slide, (8) maintaining documentation for the validation process, and (9) randomization. Following these recommendations and statements, our validation study obtained an overall intra-observer diagnostic concordance of 95.5%, above the recommended cut-off of 95% concordance. Thus, CDPPN was successfully validated. Intra-observer IHC interpretation concordance (digital slide versus glass slide) was also studied and the concordance rate for IHC was 100%.

The CAP guidelines^5^ recommended that, for any additional applications (such as IHC), an additional 20 cases should be used for validation. In our study, the IHC slides were necessary for the diagnoses. Thus, we consider IHC slides part of the primary application, not an additional application. We do not have additional application in this study. We used IHC in 24 cases creating a total of 149 valid data points and all these data points were concordant. If we were to consider the IHC tests an additional application, the numbers would have met the 20-case minimum recommended by CAP.

This study included many types of pediatric cancer. However, the majority (8 out of 12) of discrepant data points were from only 2 types of cancer: differentiating neuroblastoma (5 data points) and rhabdomyosarcoma (3 data points). The diagnosis of differentiating neuroblastoma requires Schwannian stroma <50% and differentiating neuroblasts >5%. The counting can be subjective and even expert pediatric pathologists may have different opinions when diagnosing this entity. The same can be said for subtyping rhabdomyosarcoma without comprehensive ancillary tests including molecular analysis. Thus, it is likely that the discrepancy rate in these cases was due to the intrinsic diagnostic difficulty of the cancer, not due to the technical platform. In this study all participants reviewed the same digital slides but separate glass slides, which is a potential source of bias favoring increased concordance in the digital arm of the study and might explain the observed better concordance in this group.

WSI-based nationwide digital pathology networks are rare. To the best of our knowledge, there are currently only 2 other such networks, 1 in China and the other 1 in the Netherlands.^9,10^ Our network is the first nationwide digital pathology consultation network in pediatric pathology. Both the Chinese and the Dutch networks had only limited validation study, typically involving only 1 or 2 pathologists. Our validation study was unique that it involved all pathologists/hospitals in the network, better imitating the real-world clinical environment. Following successful validation, CDPPN is now in full clinical use, serving children in Canada. We are currently working to expand the network to provide international consultations.
